# N-methyl-D-aspartate Receptors and Depression: Linking Psychopharmacology, Pathology and Physiology in a Unifying Hypothesis for the Epigenetic Code of Neural Plasticity

**DOI:** 10.3390/ph17121618

**Published:** 2024-11-30

**Authors:** Stefano Comai, Sara De Martin, Andrea Mattarei, Clotilde Guidetti, Marco Pappagallo, Franco Folli, Andrea Alimonti, Paolo L. Manfredi

**Affiliations:** 1 Department of Pharmaceutical and Pharmacological Sciences, University of Padua, 35121 Padua, Italy; stefano.comai@unipd.it (S.C.); sara.demartin@unipd.it (S.D.M.); andrea.mattarei@unipd.it (A.M.); 2 Department of Biomedical Sciences, University of Padua, 35121 Padua, Italy; 3 Department of Psychiatry, McGill University, Montreal, QC H3A 1A1, Canada; 4 IRCSS San Raffaele Scientific Institute, 20132 Milan, Italy; 5 Child Neuropsychiatry Unit, Department of Neuroscience, IRCCS Bambino Gesù Pediatric Hospital, 00165 Rome, Italy; cguidetti@mgh.harvard.edu; 6 Department of Psychiatry, Harvard Medical School, Boston, MA 02115, USA; 7 Relmada Therapeutics, Inc., Coral Gables, FL 33134, USA; mpappagallo@relmada.com; 8 MGGM LLC, 85 Baker Road, Kerhonkson, NY 12446, USA; 9 Department of Health Sciences, University of Milan, 20141 Milan, Italy; franco.folli@unimi.it; 10 The Institute of Oncology Research, Università della Svizzera Italiana, 6500 Bellinzona, Switzerland; andrea.alimonti@ior.usi.ch; 11 Veneto Institute of Molecular Medicine, 35129 Padua, Italy; 12 Department of Medicine, Zurich University, 8006 Zurich, Switzerland; 13 Department of Medicine, University of Padua, 35122 Padua, Italy

**Keywords:** AMPA, Ca^2+^, endorphins, glutamate, Mg^2+^, major depressive disorder, neural plasticity, NMDA

## Abstract

Uncompetitive NMDAR (N-methyl-D-aspartate receptor) antagonists restore impaired neural plasticity, reverse depressive-like behavior in animal models, and relieve major depressive disorder (MDD) in humans. This review integrates recent findings from in silico, in vitro, in vivo, and human studies of uncompetitive NMDAR antagonists into the extensive body of knowledge on NMDARs and neural plasticity. Uncompetitive NMDAR antagonists are activity-dependent channel blockers that preferentially target hyperactive GluN2D subtypes because these subtypes are most sensitive to activation by low concentrations of extracellular glutamate and are more likely activated by certain pathological agonists and allosteric modulators. Hyperactivity of GluN2D subtypes in specific neural circuits may underlie the pathophysiology of MDD. We hypothesize that neural plasticity is epigenetically regulated by precise Ca^2+^ quanta entering cells via NMDARs. Stimuli reach receptor cells (specialized cells that detect specific types of stimuli and convert them into electrical signals) and change their membrane potential, regulating glutamate release in the synaptic cleft. Free glutamate binds ionotropic glutamatergic receptors regulating NMDAR-mediated Ca^2+^ influx. Quanta of Ca^2+^ via NMDARs activate enzymatic pathways, epigenetically regulating synaptic protein homeostasis and synaptic receptor expression; thereby, Ca^2+^ quanta via NMDARs control the balance between long-term potentiation and long-term depression. This NMDAR Ca^2+^ quantal hypothesis for the epigenetic code of neural plasticity integrates recent psychopharmacology findings into established physiological and pathological mechanisms of brain function.

## 1. Introduction

**Figure 1:**
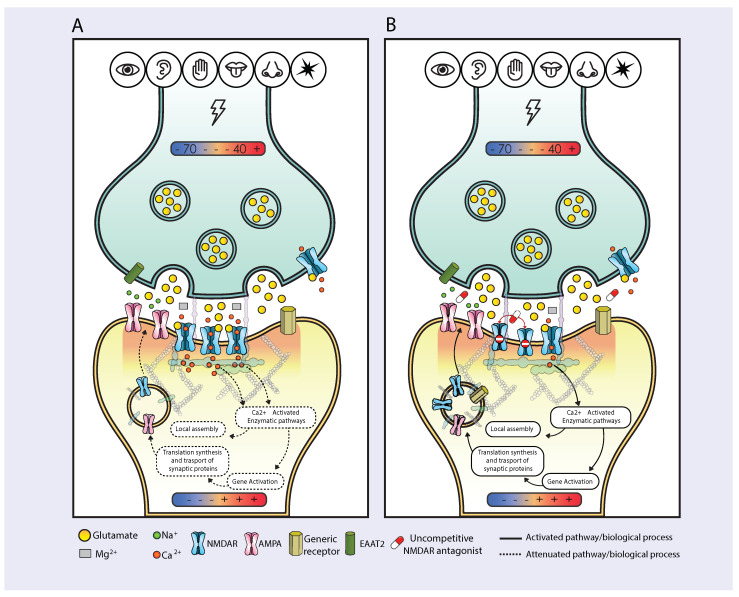
The depressive phenotype, impaired neural plasticity, and uncompetitive NMDAR antagonists: psychopharmacology of dysfunctional synapses in depression. (**A**) Receptor cell → first-order neuron synapse in patients with depression. At resting membrane potential, hyperactive postsynaptic NMDARs determine excessive Ca^2+^ influx, leading to chronic hyper-activation of CaMKIII-eEF2 signaling and downstream effectors, causing unavailability of synaptic proteins in neurons within brain circuits relevant to depression. (**B**) Low potency uncompetitive NMDAR antagonists preferentially block GluN2D channels in the open conformation and free of Mg^2+^ during resting membrane potential. The decreased influx of GluN2D-mediated Ca^2+^ restores synaptic protein homeostasis through down modulation of the CaMKIII-eEF2 pathway and re-activation of downstream effectors. Postsynaptic protein homeostasis enables physiological neural plasticity and determines the resolution of the depressive phenotype. Solid lines represent activated pathways/biological processes; dashed lines indicate attenuated pathways/biological processes.

Hansen and colleagues in their 2018 review describing the structure and function of ionotropic N-methyl-D-aspartate receptors (NMDAR) predicted that the emergence of uncompetitive NMDAR antagonists as antidepressants would advance our understanding of the role of NMDARs in physiology and disease [1]. The aim of this hypothesis paper is to integrate the discovery of the clinical effects of uncompetitive NMDAR antagonists in patients with major depressive disorder (MDD) into the current scientific body of knowledge on the pivotal role of NMDARs in neural plasticity. The discovery of the antidepressant effects of uncompetitive NMDAR antagonists in patients with MDD is providing key information for advancing the understanding of the molecular basis of the physiology and pathology of emotions and behaviors (Figure 1). Our integrative approach aims to unify seemingly separate perspectives on NMDAR function, offering a unifying framework that links the antidepressant effects of uncompetitive NMDAR antagonists with the role of NMDARs in neural plasticity. This synthesis of converging lines of evidence related to NMDARs and neural plasticity may not only further our understanding of NMDARs in the context of depression but also drive advances in neuropsychiatric pathophysiology and psychopharmacology.

MDD is the leading cause of disability worldwide, with approximately 300 million affected people (GBD 2018). In the USA, the reported lifetime prevalence for MDD is approximately 20% [2]. Of note, a steep increase in the prevalence of MDD was recorded during the COVID-19 pandemic [3], underscoring the known correlation between MDD and stressful events, including social isolation, in alignment with the known adaptive role of the depressive phenotype [4]. Because of the absence of reliable biomarkers, the distinction between transient reactive depression and MDD is not straightforward and is based on specialized guideline descriptors [5]. The depressive-like phenotype is considered a physiological response during bereavement [6,7,8] or in conjunction with other losses, such as the loss of a partner, a job, etc. Furthermore, short-lived depressive-like behaviors and feelings are not only considered normal but also expected during certain ceremonies, such as religious or similar types of gatherings. Similarly, manic-like behaviors and symptoms are often considered normal in other contexts, such as political rallies or certain sports events. The psychosocial and physiological aspects of depressive-like and manic-like behaviors suggest that these behaviors may serve evolutionary purposes. For example, during stressful events, depressive-like emotions, and behaviors, such as lack of initiative and motivation, may represent species-preserving behaviors; manic-like behaviors could instead be advantageous in competitive situations. Although the definition of “normality” in relation to behaviors and emotions depends on psychosocial settings, which vary geographically and over time, individuals typically exhibit “balanced” behaviors and emotions within a spectrum of cultural settings [9,10]. However, when depressive-like behaviors and depressive emotions are severe, prolonged, or become relatively independent of stressful triggers, a diagnosis of MDD may be made [5]. Stressful conditions are used in experimental models of depressive-like behavior, and these models can predict the potential efficacy of antidepressant drug candidates, including uncompetitive NMDAR antagonists [11,12,13].

The monoamine-based hypothesis of depression was proposed over half a century ago and has since dominated the field, leading to the worldwide widespread clinical use of monoaminergic antidepressants. However, the glutamatergic system, which plays a key role in memory and cognition [1,14], may also be pivotal for regulating mood and emotions [15,16,17,18]. As a result, certain mood disorders may primarily originate from dysregulation within the glutamatergic system. The initial observation of the antidepressant actions of ketamine [19] has paved the way for the development of uncompetitive NMDAR antagonists, including esketamine and the combination drug dextromethorphan-bupropion, as a promising new class of antidepressants [13,20,21,22,23,24,25,26,27,28,29,30]. The highlights of the prevailing hypothesis for the MOA of NMDAR antagonist antidepressants [13,23,24,25,26,27,28,29,30] are illustrated in Figure 1. Esmethadone (REL-1017) is another activity-dependent uncompetitive antagonist of the NMDAR, currently in advanced clinical development [20,31,32].

The innovation in antidepressant treatment by uncompetitive NMDAR antagonists was sparked by the discovery that ketamine, a dissociative anesthetic and racemic mixture of (R)-ketamine and (S)-ketamine, induces rapid antidepressant effects [19,33]. While both enantiomers of ketamine are NMDAR antagonists, only the more potent S-enantiomer, esketamine, has received FDA approval as an intranasal formulation. The adverse effects of intranasal esketamine, such as short-lived dissociative effects in over 70% of patients, require inpatient clinical observation following administration [34]. Consequently, the FDA has limited its marketing approval to patients with TRD and MDD with acute suicidal ideation or behavior.

At first, the “disinhibition hypothesis” attempted to explain the antidepressant effects of ketamine within the paradox of enhanced neural plasticity triggered by a dissociative agent that blocks the NMDAR [35]. In contrast with earlier assumptions, there is now evidence that the induction of dissociative effects may not be necessary for the rapid antidepressant effects of ketamine and its enantiomers [36]. The lack of correspondence between dissociative effects and antidepressant effects with uncompetitive NMDAR antagonists was further supported by the observation that the combination dextromethorphan-bupropion determines antidepressant effects in the absence of dissociative effects [37,38]. This lack of correspondence between dissociative effects and antidepressant effects was also observed in clinical trials testing esmethadone [31,32].

The current prevailing hypothesis for the MOA of uncompetitive NMDAR antagonists in MDD builds on prior work on the role of graded Ca^2+^ influx via NMDARs at resting membrane potential in regulating synaptic protein homeostasis [23,39,40]. The “homeostatic hypothesis” suggests that the activity-dependent uncompetitive block of tonically and pathologically hyperactive NMDARs leads to the restoration of neural plasticity [27], has progressively gained acceptance [41,42]. This hypothesis also includes the preferential block of GluN2D subtypes by uncompetitive NMDAR antagonists [28,43] (Figure 1). The “homeostatic hypothesis” explains the MOA of uncompetitive NMDAR antagonists in depression and also implies a mechanism of disease (MOD) for MDD that integrates the central role of NMDARs in neural plasticity. According to this MOD hypothesis, MDD is caused by chronic low-level excitotoxicity due to tonic NMDAR hyperactivity and excessive Ca^2+^ influx at graded resting neuronal membrane potential in circuits relevant for MDD. Since uncompetitive NMDAR antagonists are activity-dependent blockers, they preferentially target pathologically open NMDAR channels, while having no significant activity at NMDAR channels in the closed conformation, which are blocked by Mg^2+^, the physiological NMDAR blocker [28,29].

A key point of convergence between the “disinhibition hypothesis” and the “homeostatic hypothesis” is that, in both hypotheses, the restoration of neural plasticity is dependent on brain-derived neurotrophic factor (BDNF) [11,29,35]. Additionally, uncompetitive NMDAR antagonist antidepressants have been shown to restore structural and functional synaptic proteins necessary for neural plasticity [11,13,23,28], such as subunits of NMDARs and subunits of α-amino-3-hydroxy-5-methyl-4-isoxazolepropionic acid receptors (AMPARs), and scaffolding proteins [1,44].

For completeness, we will briefly discuss other MOA hypotheses for the antidepressant actions of uncompetitive NMDAR antagonist antidepressants. These alternative MOA hypotheses suggest that the antidepressant effects of these drugs may be explained by mechanisms including mu opioid receptor (MOR) agonism, sigma 1 receptor binding, and serotonin receptor activation. Some of these proposed hypotheses for the non-NMDAR-mediated antidepressant mechanisms of drugs or candidate drugs classified as uncompetitive NMDAR antagonists [45,46] are discussed further in other sections of this review and have been discussed in other publications [32,47]. However, of note, recent studies [29] confirm the earlier work, indicating that the most likely MOA for the antidepressant effects of ketamine is the activity-dependent uncompetitive block of hyperactive ionotropic NMDARs in neurons part of circuits relevant for MDD [11,13,23].

## 2. The Glutamatergic System

**Figure 2:**
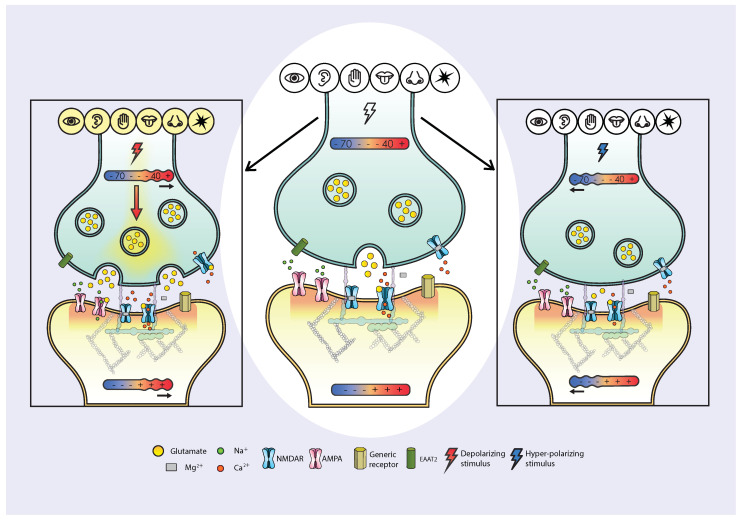
Physiological stimulus-driven NMDAR-mediated Ca^2+^ regulated neural plasticity at resting and at phasic membrane potential. The figure depicts a receptor cell → first-order neuron synapse following depolarizing or hyper-polarizing stimuli. At resting membrane potential, subthreshold stimuli are reaching the receptor cell (center figure) with tonic release of glutamate, activating a small fraction of AMPARs and NMDARs, resulting in preferential GluN2D graded postsynaptic influx of Ca^2+^ quanta. Tonic NMDAR-mediated graded Ca^2+^ influx (preferentially via GluN2D subtypes) directs synaptic protein homeostasis. When depolarizing stimuli reach the receptor cell (left figure), there is a massive release of glutamate into the synaptic cleft. This release activates all postsynaptic ionotropic receptors, e.g., ”fast” Na^+^ permeable AMPARs“and ”slow” Ca^2+^ permeable NMDARs. For completeness, we also show the response to stimuli resulting in receptor cell hyperpolarization (right figure), e.g., visual stimuli reaching photoreceptors, with a reduction in the tonic release of glutamate. Reduced glutamate release leads to a graded reduction in NMDAR-mediated Ca^2+^ entry into the postsynaptic neuron.

Glutamate is the key excitatory neurotransmitter in the central nervous system. Presynaptic stimulus-triggered glutamate release activates NMDAR-regulated postsynaptic Ca^2+^ influx [1] (Figure 2).

Glutamate acts at ionotropic (NMDA, AMPA, and kainate receptors) and metabotropic (mGlu) receptors. Ionotropic glutamate receptors are channels formed by four protein subunits that delimit a pore through which there is selective passage of ions. NMDARs are “slow” receptors that open for a subtype-determined amount of time (from 50 ms to a few seconds, depending on subunit composition). NMDARs are selectively permeable to Ca^2+^ and are physiologically blocked by Mg^2+^. As far as their structure is concerned, NMDARs are composed of two glycine-binding GluN1 subunits, necessary for the membrane expression of the functional receptor, and two glutamate-binding GluN2 subunits. There are four GluN2 isoforms, i.e., GluN-2A, 2B, 2C, and 2D. Each subunit has distinct regional and developmental distribution in the brain. NMDARs containing subunits 2A, 2B, 2C, and 2D regulate Ca^2+^ influx across the neuronal membrane during phasic (action potential) and tonic (graded resting membrane potential) receptor activity, differentially according to subunit composition [48,49,50].

NMDAR Ca^2+^ influx at resting membrane potential, similar to Ca^2+^ influx during an action potential, is regulated by agonists and allosteric modulators under both physiological and pathological settings [1,51,52]. Glutamate binding to its site on the GluN2 subunit induces a conformational change in the NMDAR, transitioning it to an “open” state. Each NMDAR requires the binding of four neurotransmitter molecules to switch the channel from “close” to “open” conformation: two glutamate molecules (one for each GluN2 subunit) and two glycine molecules (one for each GluN1 subunit). Furthermore, NMDARs are physiologically blocked by Mg^2+^, which occupies the channel pore of the majority of NMDARs at resting membrane potential [1,52]. Free glutamate released into the synaptic cleft by the presynaptic neuron binds not only to NMDARs but also to AMPAR, specifically 2A subunits, briefly opening the “fast” AMPAR channel (Figure 2) for approximately 1 ms. AMPARs containing the 2A subunit are primarily permeable to Na^+^, and their opening allows a rapid influx of Na^+^, leading to membrane depolarization. As the membrane potential shifts from around −85 mV towards −55 mV (the approximate range for a graded resting membrane potential), the probability of Mg^2+^ disengaging from the NMDAR channel pore increases gradually. When the NMDAR is in the open conformation (i.e., bound by glutamate and glycine) and free of Mg^2+^, the channel pore becomes permeable to Ca^2+^ for a subtype-specific duration.

Excessive and prolonged presence of free glutamate in the synaptic cleft (Figure 1; ambient glutamate) leads to excessive Ca^2+^ influx into the postsynaptic cell, potentially triggering excitotoxicity. This condition may result in varying degrees of cellular dysfunction, including neuronal death [53,54,55,56]. Excitotoxicity can be either chronic (e.g., MDD and other recurring or chronic neuropsychiatric disorders) or acute (e.g., post-trauma or post-stroke). Patients with MDD display reduced hippocampal volume, suggesting neuronal loss [57]. Both preclinical and clinical studies indicate a link between neuronal loss and depression [58].

In summary, MDD may be caused by chronic excitotoxicity resulting from the hyperactivation of ionotropic NMDARs, preferentially involving the GluN2D subtype, expressed by neurons part of MDD-relevant circuits. This hyperactivation leads to downregulation of synaptic proteins, impairing neural plasticity in specific neural circuits (Figure 1) [28]. The “homeostatic hypothesis” aligns with recent in vivo findings indicating that ketamine blocks NMDARs in a brain region–and depression state–specific manner [29].

Metabotropic glutamate receptors (mGluRs), activated by glutamate, initiate G-protein-coupled signaling cascades that regulate intracellular calcium and second messengers, rather than directly mediating ion flux. Among them, mGluR5, a Group I mGluR co-localized with NMDARs, plays a key role in modulating NMDAR activity. Although a detailed discussion of mGluRs is beyond the scope of this NMDAR-focused paper, it is important to note that mGluR5 signaling can influence NMDAR-mediated Ca^2+^ signaling, thus contributing to Ca^2+^-dependent neural plasticity [59].

## 3. Neural Plasticity: Decoding and Integrating External Stimuli into Functional Afferent and Efferent Neural Circuits

The brain is composed of neurons, which serve as the primary units for memory coding and are interconnected via synapses. Beyond neurons, the neural network includes other critical cell types. Astrocytes play multifaceted roles, including the regulation of the blood–brain barrier (BBB) to maintain the brain’s microenvironment. They also facilitate the recycling of neurotransmitters, including glutamate, through specific transport mechanisms and metabolic pathways. Oligodendrocytes contribute to efficient signal propagation by producing myelin sheaths that insulate neuronal axons. Microglia act as the immune cells of the brain, scavenging cellular debris, providing structural support, and participating in immunological responses [60,61,62].

Consistent with a unified hypothesis, NMDAR-mediated Ca^2+^ signaling and susceptibility to excitotoxicity have also been observed in astrocytes [63], microglia [64], and oligodendrocytes [65,66].

**Figure 3:**
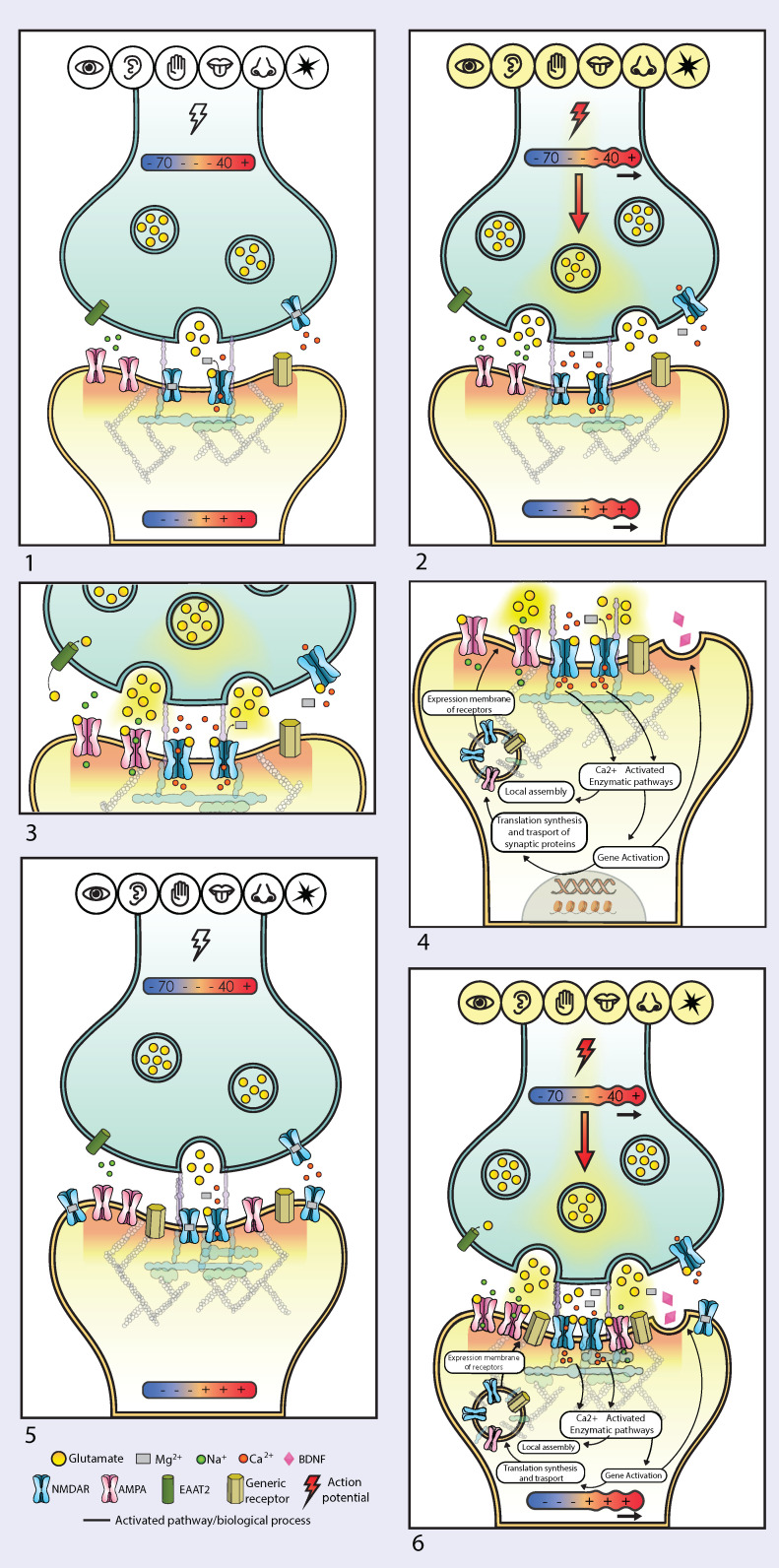
Stimulus-induced NMDAR-regulated Ca^2+^ quanta and “hot spot” neural plasticity (**1**–**6**) receptor cell → first-order synaptic neuron. (**1**) Resting membrane potential: Stimuli reach the receptor cell and change its membrane potential, regulating the activity of presynaptic NMDARs. Based on the frequency and intensity of incoming stimuli, graded Ca^2+^ quanta via presynaptic NMDARs instruct the receptor cell on glutamate vesicle density. When presynaptic glutamate vesicles reach the density threshold, one or more vesicles fuse with the membrane of the receptor cell and release glutamate. Free glutamate released at graded resting membrane potential activates a small percentage of glutamate receptors, including AMPARs and NMDARs, at the “hot spot” of the first order neuron. The low nanomolar concentration of free glutamate preferentially activates GluN2D subtypes. (**2**) Action potential: When depolarizing stimuli reach the receptor cell, release of glutamate occurs from all vesicles juxtaposed to the membrane, leading to massive activation of “hot spot” postsynaptic glutamate receptors, including AMPARs and NMDARs. (**3**) Coincidental AMPAR-mediated depolarization (“fast” Na^+^ influx) causes Mg^2+^ release from open-conformation NMDARs bound by glutamate, initiating NMDAR-regulated “slow” Ca^2+^ influx. Excitatory Amino Acid Transporters (EAATs) re-uptake free glutamate from the synaptic cleft, terminating the action potential. (**4**) NMDAR-regulated postsynaptic Ca^2+^ quanta activate enzymatic pathways, leading to the transcription of genes encoding synaptic proteins. Synthesized proteins are then transported (trafficking) to the synapse and undergo local assembly and expression at the synaptic membrane (receptor subunits and scaffolding proteins) or are released in the synaptic cleft (neurotrophic factors). (**5**) After depolarizing stimuli, EAAT activity restores resting membrane potential. Subthreshold stimuli at resting membrane potential again induce graded glutamate release, and graded activation of AMPARs and NMDARs, leading to preferential GluN2D-regulated postsynaptic Ca^2+^ influx, instructing synaptic protein homeostasis. Stimuli induce neural plasticity at the synaptic “hot spot”: the synaptic framework (type and density of receptors) and availability of synaptic proteins (receptor subunits, neurotrophic factors, and scaffolding proteins) are regulated by incoming stimuli or lack of thereof (points 2–4). (**6**) Subsequent tonic or depolarizing stimuli trigger graded or massive glutamate release into the synaptic cleft, activating AMPARs and NMDARs, directing graded or massive NMDARs influx of Ca^2+^ quanta, respectively, and downstream enzymatic activation. Free glutamate is then re-up-taken via EAATs. The epigenetic code, how keys change locks: after each stimulus, Ca^2+^ quanta and intracellular enzymatic pathways activation will be different compared to before the stimulus, due to stimulus-induced changes in the “hot spot” receptor framework. The constant stimulus-dependent changes in the receptor framework will determine NMDAR-regulated Ca^2+^ quanta influx, constantly accounting for prior stimuli. The synaptic framework at the “hot spot” precisely gates glutamate-induced NMDAR Ca^2+^ quanta influx and downstream events constantly shaping LTP/LTD in neural circuits relevant to incoming stimuli.

Neurons are connected in circuits by synapses [67]. Stimuli induce neurons to form and refine synapses, connecting sensory and motor pathways into functional circuits [68]. Synapses form and strengthen with repeated activation: neurons that fire together wire together [69]. Neural plasticity is the dynamic balancing between long-term potentiation (LTP) and long-term depression (LTD). LTP is a form of activity-dependent plasticity resulting in a persistent enhancement of synaptic transmission, whereas LTD is its complementary process, in which the efficacy of synaptic transmission is reduced [70]. Neural circuits relevant to species survival are continuously formed and refined (LTP/LTD balance) by stimuli (or lack of stimuli) from the environment (Figure 2). LTP and LTD continuously modify the structure of functional circuits with preferential integration of experiences advantageous for species-preserving prediction-based activities. Frequency and intensity of environmental stimuli reaching the detection threshold at receptor cells (e.g., photoreceptors, cochlear and vestibular hair cells, skin mechanoreceptors, olfactory and gustatory cells) determine a graded change in their membrane potential: the membrane potential may increase (e.g., cochlear cells) or decrease (photoreceptors) (Figure 1, Figure 2 and Figure 3). The understanding of the pivotal role of NMDARs in LTP and LTD via Ca^2+^ signaling spans several decades of collective work [14]. The stimulus-regulated change in membrane potential described above and illustrated in Figure 1, Figure 2 and Figure 3 results in glutamate release and cellular influx of NMDAR-mediated quanta of Ca^2+^. The number of quanta of Ca^2+^ influx via NMDARs is determined presynaptically by the density of glutamate vesicles and postsynaptically by the framework of glutamatergic receptors expressed on the membrane of cells (Figure 1, Figure 2 and Figure 3).

The evolutionary purpose of memorizing experiences by forming structural and functional neural circuits is to attempt to predict future incoming environmental stimuli and events (sensory neural circuits) while efficiently (in real-time) implementing constantly upgraded patterns of bodily activities (motor neural circuits), both somatic and autonomic, that enhance the probability of organism survival. These integrated sensory and motor circuits are constantly strengthened or weakened through LTP or LTD, respectively, based on the frequency and intensity of incoming environmental stimuli [70]. Neural plasticity has been defined as “the ability of the nervous system to change its activity in response to intrinsic or extrinsic stimuli by reorganizing its structure, functions, or connections” [71]. Thus, neural plasticity consists of the constant lifetime dynamic balancing of two complementary forces, LTP and LTD, constantly forming and refining integrated afferent and efferent circuits. Connections between neurons are constantly enhanced or weakened in a dynamic interface between the organism and its environment aimed to select and maintain integrated sensory and motor circuits that enhance species survival. The first example of a functional stimulus-generated integrated sensory and motor circuit was described by Pavlov in 1927 [68]. A century of collective work can now attempt to illustrate the molecular mechanism of integrated sensory and motor neural plasticity (Figure 1, Figure 2 and Figure 3).

The key molecules in NMDAR-mediated Ca^2+^ signaling are Ca^2+^/calmodulin-dependent protein kinase II (CaMKII), calcineurin, and CaMKIII-eEF2 kinase. When Ca^2+^ enters the postsynaptic neuron via NMDARs, it binds to calmodulin, a calcium sensor protein. The Ca^2+^-calmodulin complex activates CaMKII, which then phosphorylates target proteins that enhance LTP, including via phosphorylation of the AMPA receptor subunit GluA1. CaMKII activated by Ca^2+^ sustains synaptic changes over time, contributing to the stability of LTP [72].

Similar to CaMKII, calcineurin is activated by the Ca^2+^-calmodulin complex after calcium influx through NMDARs. Once activated, calcineurin dephosphorylates proteins associated with the AMPA receptor, such as stargazin, leading to the internalization of AMPA receptors from the synaptic membrane, thereby weakening synaptic transmission and enhancing LTD. LTP and LTD are finely balanced by the activities of CaMKII and calcineurin. While CaMKII drives synaptic strengthening (LTP), calcineurin mediates synaptic weakening (LTD). This dynamic regulation allows neurons to adjust the strength of synaptic connections in response to activity [73].

Finally, the CaMKIII-eEF2 kinase pathway may be the key target of the activity of uncompetitive NMDAR antagonists used as antidepressants. CaMKIII-eEF2 kinase regulates synaptic protein synthesis. When calcium enters the neuron via NMDARs, it activates eEF2 kinase through the Ca^2+^-calmodulin complex. eEF2 kinase then phosphorylates eEF2, leading to the inhibition of synaptic protein translation. Excessive inhibition of synaptic protein translation may be the molecular basis of MDD corrected by uncompetitive NMDAR antagonists Under normal conditions, the activation of eEF2 kinase can slow down the overall rate of protein synthesis, allowing for fine-tuning of protein production that is crucial for structural changes at the synapse. NMDAR antagonists for MDD, such as ketamine, reduce the excessive phosphorylation of eEF2, promoting the synthesis of synaptic proteins, such as NMDAR subunits and BDNF, which are critical for synaptic growth and strengthening [28].

Calcium-induced calcium release (CICR) is another important contributor to calcium signaling. Once calcium enters the neuron, it can trigger the release of more calcium from intracellular stores, like the endoplasmic reticulum (ER) through ryanodine receptors (RyRs) or IP3 receptors (IP3Rs). This amplifies the calcium signal through the cytoplasm. CICR may be more relevant for voltage-gated calcium channels (VGCCs) than for NMDARs [74,75].

While CICR and mGluR5 can modulate NMDAR-mediated Ca^2+^ signaling and may contribute to the amplification and broader regulation of calcium signaling within the neuron, the primary activity of NMDAR-mediated Ca^2+^ signaling occurs within the intracellular domain of the NMDAR complex itself. Ca^2+^ entering through NMDARs directly activates CaMKII, calcineurin, and eEF2 kinase, all of which are crucial for LTP, LTD, and synaptic protein synthesis regulation. This highly localized calcium signaling at the synapse is what makes NMDARs central to activity-dependent neural plasticity necessary for the adaptive synaptic changes as the basis of learning and memory.

As anticipated by Hansen and colleagues in 2018, the emergence of uncompetitive NMDAR antagonists as antidepressants is advancing the understanding of the role of NMDARs in physiology and disease [1]. As already mentioned, the MOD hypothesis for MDD is shifting away from the classic serotonergic hypothesis [76]. Recent advancements in psychopharmacology suggest that impaired neural plasticity, resulting from dysregulated glutamatergic Ca^2+^ signaling via NMDARs, may play a pivotal role in the depressive phenotype [1,15,16]. Indeed, preclinical models have demonstrated that the antidepressant-like activity of NMDAR uncompetitive antagonists is dependent on the reversal of impaired spinogenesis [11,23,28,41,42].

## 4. NMDAR Psychopharmacology and the Epigenetic Code Hypothesis

The molecular mechanisms at the basis of the MOA of uncompetitive NMDAR antagonist antidepressants provide the basis for a glutamatergic NMDAR-centered MOD for MDD (Figure 1) [28]. This MOD based on NMDAR subtype-specific hyperactivity at graded resting membrane potential seeks to explain physiology (e.g., reactive depressive behaviors) and pathology (MDD and other disorders caused by chronic excitotoxicity) with a common molecular theory of stimulus-induced downregulation of the LTP component of neural plasticity. The reduced availability of synaptic proteins, caused by hyperactivity of NMDARs at resting membrane potential, downregulates LTP [28].

Stimulus-dependent variations in presynaptic glutamate release influence the postsynaptic influx of Ca^2+^ ions through NMDARs, with graded Ca^2+^ quanta release at resting membrane potential and massive Ca^2+^ quanta release during action potential. This influx not only plays a central role in circuit connectivity by regulating LTP and LTD but also dynamically modifies the synaptic framework (ensemble of receptors at synapses) (Figure 3). The levels of NMDAR subunits and the expression of NMDAR subtypes change, following memory acquisition in vivo [77]. In vitro studies show that uncompetitive NMDAR antagonists modify NMDAR subunit expression [78] whereas, in vivo studies show that they enhance synaptic spines and increase BDNF in vivo, while reversing depressive-like behavior [11,13]. Thus, the influx of Ca^2+^ quanta through NMDARs influences downstream events that regulate the synaptic framework, including the expression of AMPARs and NMDARs at the “hot spot”, the 100–200 nm area on the membrane of second-order neurons juxtaposed to presynaptic glutamate release (Figure 3). The “keys” (Ca^2+^ quanta via NMDARs) continuously modify their own “keyholes” (NMDARs and AMPARs expressed on the membrane) through downstream effects that regulate translation, transport, assembly, and the expression or release of synaptic proteins, including AMPARs and NMDARs subunits, as well as neurotrophic factors. Therefore, the configuration of AMPAR and NMDAR receptor frameworks at any given moment at the synaptic “hot spot” and in related areas (e.g., extra-synaptic receptors, including auto-receptors) is dynamically modified by NMDAR-mediated Ca^2+^ quanta influx.

As already mentioned, NMDARs are tightly regulated channels that require two ligands, glutamate and glycine. Moreover, they are also uniquely regulated by various endogenous and exogenous positive allosteric modulators (PAMs) and negative allosteric modulators (NAMs) [1,51]. When in an open conformation, NMDARs are selectively permeable to Ca^2+^. However, Mg^2+^, a physiological blocker of NMDAR, must first disengage from the channel pore before Ca^2+^ can enter the cell from the extracellular space. The likelihood of Mg^2+^ disengagement depends on membrane polarization, which is primarily influenced by glutamate action at AMPARs (Figure 3). As the membrane potential moves from −85 mV to −55 mV, the probability of Mg^2+^ disengagement from the NMDAR pore increases due to the rapid Na^+^ influx associated with the brief (1 ms) opening of AMPAR by glutamate. Consequently, glutamate in the synaptic cleft not only activates AMPARs causing membrane depolarization and facilitating Mg^2+^ disengagement from the NMDAR pore, but also triggers NMDAR activation through allosteric changes induced by glutamate binding to N2 subunits. NMDAR-mediated Ca^2^+ signaling initiates a cascade of biochemical events involving interaction with calcium-binding proteins, activation of calcium-dependent enzymes, and modulation of transcription factors, finally leading to changes in the gene transcription within the nucleus. Therefore, the epigenetic code instructing genes controlling neural plasticity consists of specific quanta of Ca^2+^ entering the neuron via NMDARs. These NMDAR-regulated quanta of Ca^2+^ activate enzymatic pathways that maintain homeostatic availability of synaptic proteins essential for translation, transport, assembly, and membrane expression of receptors at the synaptic “hot spot”, thereby modulating synaptic connectivity. In summary, stimulus-induced NMDAR-mediated Ca^2+^ signaling results in specific patterns of gene expression.

The number of Ca^2+^ quanta entering the postsynaptic neuron via NMDARs is determined by presynaptic glutamate release, which is regulated by specialized stimulus-transducing receptor cell (Figure 1, e.g., a skin mechanoreceptor or an auditory hair-receptor cell). Glutamate release can be tonic, during resting membrane potential (when non-depolarizing stimuli are reaching the presynaptic neuron and only a small percentage of NMDARs are Ca^2+^ permeable), or phasic, at action potential, triggered by depolarizing stimuli, leading to massive activation of NMDARs. While the “slow” Ca^2+^ permeable NMDARs regulate neural plasticity via downstream Ca^2+^ effects initiated within the intracellular portion of the receptor, within the postsynaptic density, “fast” Na^+^ permeable AMPARs modulate NMDAR activity. Indeed, AMPARs, through glutamate-mediated changes in membrane potential, act as an “enhancer” for NMDAR activity by facilitating the disengagement of Mg^2+^ from the NMDAR pore. The quanta of Ca^2+^ entering the postsynaptic neuron via NMDARs are precisely regulated by the dynamic postsynaptic receptor framework, including AMPARs. AMPARs influence (dimmer/enhancer) NMDAR Ca^2+^ entry indirectly by inducing short-lived (1 ms) Na^+^-dependent depolarizations, causing Mg^2+^ disengagement from the NMDAR channel pore. This regulation occurs during both tonic (−85 mv to −55 mv) and phasic (−55 mv towards positive) changes in membrane potential. NMDARs, on the other hand, directly control Ca^2+^ quanta influx through a tightly regulated subtype-specific open time.

In summary, NMDAR activity modulates the influx of Ca^2+^ quanta, which in turn activate enzymatic pathways that epigenetically influence structural changes at the synaptic framework, particularly at the synaptic “hot spot” throughout the lifetime of the organism (Figure 3). These ongoing structural changes at the “hot spot” underpin the dynamic nature of neuronal circuits at the basis of neural plasticity. This dynamic modulation at the “hot spot” allows neuronal circuits to continuously adapt to environmental changes, with physiological mechanisms like LTP and LTD, enabling real-time adjustments of organisms’ responses to detectable stimuli generated by the surrounding environment.

Excitatory amino acid transporters (EAATs) continuously remove glutamate from the synaptic cleft, thereby reducing the proportion of NMDARs and AMPARs in the open conformation. The resting membrane potential is graded and dynamic, depending significantly on the frequency and number of glutamate vesicles that fuse with the membrane of the presynaptic neuron, leading to glutamate release into the synaptic cleft. The intensity and frequency of incoming stimuli reaching the receptor cell modulate its membrane potential, influencing NMDAR-mediated Ca^2+^ influx and determining the density of intracellular glutamate vesicles [79]. The stimulus-dependent density of glutamate vesicles within the receptor cell affects the probability of vesicle fusion with the membrane, resulting in glutamate release. The removal of glutamate from the synaptic cleft by Na^+^-driven EAATs not only reduces the availability of glutamate for binding to the GluN2 subunit of NMDARs but also decreases the glutamate available for AMPARs binding. This reduction in AMPAR-mediated Na^+^ influx drives the membrane potential towards more negative values, facilitating the engagement of Mg^2+^ within the NMDAR channel pore. However, it is important to note that different NMDAR subtypes have varying affinities for Mg^2+^: GluN2D subtypes, for instance, have low affinity for Mg^2+^ and the longest opening time [1,80], making it more likely to remain open even when glutamate levels in the synaptic cleft are in the low nanomolar range [43]. The preferential activation of GluN2D subtypes by low-level glutamate makes them the most likely target for activity-dependent uncompetitive NMDAR antagonists.

The type, frequency, and intensity of specific environmental stimuli determine the density of glutamate vesicles at the presynaptic neuronal area juxtaposed to the postsynaptic “hot spot”. The “hot spot” is the glutamate receiving area of 100–200 nm in diameter of the postsynaptic neuron. As glutamate vesicles accumulate in the presynaptic neuron, the probability of vesicle fusion with the presynaptic membrane increases. When one or more presynaptic glutamate vesicles fuse with the membrane, they release glutamate in the synaptic cleft and activate the glutamate receptors present in the “hot spot”. Glutamate release from the presynaptic neuron can be massive, an all-or-none phenomenon, when the membrane potential reaches the action potential threshold (approximately −55 mV). It can also be graded during the periods between action potentials when the membrane is at the resting potential (approximately −85 to −55 mV) (Figure 2). The “hot spot” contains various receptors, including AMPARs and NMDARs. The NMDAR channel pore opens when the receptor undergoes a conformational change and when Mg^2+^ disengages from the channel pore. The opening of the NMDAR channel allows a subtype-specific time-controlled influx of Ca^2+^ into the postsynaptic neuron. This tightly regulated Ca^2+^ influx through NMDARs is a key factor for the formation of neural circuits that underly memory formation. Therefore, memory (neural plasticity) is instructed by quanta of Ca^2+^ via NMDARs. These Ca^2+^ quanta, regulated by specific NMDAR subtypes, activate selected downstream enzymatic pathways within the intracellular portion of NMDARs [1].

In the postsynaptic neuron, during resting membrane potential, Ca^2+^ triggered downstream pathways guide gene expression, determining the type and quantities of synaptic proteins necessary for the LTP and LTD of the synapse over time (ongoing neural plasticity). LTP, the most studied form of synaptic plasticity, is associated with memory formation and is structurally visible with high-resolution imaging techniques as an increase in synaptic spine size and density. Conversely, LTD leads to a reduction in synaptic spine size and density, weakening or eliminating less active synaptic connections and neuronal circuits.

The evolutionary purpose of pruning is to refine and optimize neural circuits, making the brain more efficient by preferentially retaining stimulus-driven connections. According to our unifying hypothesis, at the molecular level, pruning, as part of the LTP/LTD balance, is regulated by the influx of Ca^2+^ quanta through NMDARs.

Physiologically, maintaining a balance between LTP and LTD is crucial to prevent synaptic saturation, which could occur during continuous circuit stimulation, if only synaptic strength were increased [81]. Glutamate and ionotropic glutamate receptors are the key players in the formation of LTP and LTD [82]. The LTP/LTD relationship is not a dichotomy but rather an equilibrium determined by the status of NMDARs. Ca^2+^ ions entering the cell at resting membrane potential, preferentially via GluN2D subtypes at low levels of synaptic glutamate, act as a signaling code, activating enzymatic pathways in the intracellular portion of the NMDAR. This downstream signaling epigenetically instructs the cell on homeostatic synaptic protein availability, by determining the types and amounts of proteins necessary to modulate individual synapses in relation to incoming stimuli. Synapses are formed and modified (via LTP and LTD) in response to the type, frequency, and intensity of external stimuli (Figure 2). A miniature presynaptic event (mPSE) occurs when a single glutamate vesicle fuses with the presynaptic membrane juxtaposed to the postsynaptic “hot spot”, releasing glutamate into the synaptic cleft during resting membrane potential. The frequency of these mPSEs, and consequently the concentration of glutamate in the synaptic cleft, governs the degree of NMDAR-mediated Ca^2+^ influx into the postsynaptic neuron, generating miniature postsynaptic currents (mPSCs).

Synaptic modulation in neural circuits can rapidly expand and interconnect spatially when neurons from different circuits are activated simultaneously [83].

The graded activity of NMDARs at resting membrane potential is essential for ensuring the homeostatic availability of synaptic proteins [23]. Without adequate synaptic proteins, efficient LTP, the encoding of new functional circuits, and effective memory formation are compromised, potentially leading to the MDD phenotype, characterized by cognitive impairment, lack of motivation, and rumination. Simultaneously, unbalanced LTD continues, potentially leading to the loss of synaptic connections potentially relevant for the individual. This imbalance can result in pathological conditions ranging from moderate MDD (mild excitotoxicity) to severe MDD, e.g., pseudodementia. In extreme cases, LTD can lead to neuronal loss, causing permanent loss of functional neural circuitry, potentially progressing to forms of excitotoxic dementia.

Synaptic proteins may include proteins that are directly related to the constant Ca^2+^ quanta-regulated balance between LTP and LTD, such as NMDAR subunits (GluN1, GluN2A, GluN2B, GluN2C, GluN2D, GluN3A, GluN3B, and their splice variants), AMPAR subunits, adhesion proteins, scaffolding proteins, and BDNF [84]. Additionally, synaptic proteins include a variety of other proteins that serve as building blocks for other receptor types expressed at the synaptic “hot spot”. Among these other receptors, GABA receptors are of particular interest.

Central nervous system (CNS) activity is often described as a balance between excitatory and inhibitory signals. While this characterization holds true, emerging evidence suggests that inhibitory activity in the brain is primarily reactive to incoming excitatory stimuli. This principle extends to other receptors present on neurons, where the translation, production, transport, assembly, and membrane expression of these receptors are regulated by NMDAR-mediated Ca^2+^ influx, which is triggered by presynaptic glutamate release in response to excitatory stimuli. In fact, to our knowledge, with the exception of photons and certain mechanical stimuli during the “release phase” after stimulation, there are no naturally occurring hyperpolarizing stimuli, i.e., stimuli able to hyperpolarize receptor cells. Even though photons and mechanical stimuli can hyperpolarize receptor cells under certain circumstances, they, like the more commonly studied depolarizing stimuli, also regulate structural neural plasticity by driving the formation of functional, stimulus-dependent circuitry [85,86] (Figure 2).

In the case of “pathological” NMDAR activity at resting potential (e.g., excessive or insufficient activity leading to altered mPSEs and mPSCs with consequent altered amount of postsynaptic Ca^2+^ influx), there may be a halting of synaptic protein translation, synthesis, transportation, assembly, and membrane expression, with the final result of unbalanced LTP and LTD (Figure 1). “Pathological” NMDAR activity at resting membrane potential can manifest as insufficient activity, as observed in scenarios like sensory deprivation, where the “normal” flow of incoming stimuli is blocked. For example, in experimental models of dark rearing, the absence of relevant circuitry selection can be observed [87]. Notably, circuitry selection can be rescued by exposure to light and even by BDNF [88].

The concept of “inadequate” external stimulation may be applied to various types of stimuli, encompassing those relevant to parental or peer bonding and even stimuli related to scholastic education.

As already mentioned, NMDAR activity at resting potential can be pathologically excessive, as observed in certain neuropsychiatric disorders including MDD [28]. The term excitotoxicity was first coined to describe the pathological changes occurring in cells as a consequence of excessive Ca^2+^ influx. Acute excitotoxicity, such as excitotoxicity caused by stroke or trauma, leading to massive glutamate spillage into the extracellular space, has been associated with neuronal death in the area surrounding the core lesion (penumbra). In experimental models of acute excitotoxicity, uncompetitive NMDAR antagonists have been shown to reduce damage in the penumbra [89,90]. Beyond acute neurotoxicity, excessive Ca^2+^ influx via NMDARs can also lead to chronic excitotoxicity, which refers to altered cellular function that may result in structural changes in the brain and contribute to neuropsychiatric disorders [48,54,91]. Chronic excitotoxicity can disrupt the balance between LTP and LTD. If homeostatic levels of synaptic proteins, which can be considered the building blocks for memory, are present in inadequate amounts in the postsynaptic density, new memory formation (i.e., synaptic framework modifications and consequent new circuits) cannot occur, because the structural elements necessary for LTP are unavailable.

Incoming sensory stimuli regulate the synthesis and packaging of glutamate in presynaptic cell vesicles as an essential first step in neurotransmission. In addition to glutamatergic receptors, other brain receptor/neurotransmitter systems (e.g., GABAergic, monoaminergic, cholinergic systems) as well as peptide neurotransmitters (e.g., nerve growth factors, endorphins, and oxytocin) and their respective receptors, are also influenced by the glutamatergic system, via NMDAR Ca^2+^ influx and downstream signaling [92,93]. Although these glutamatergic-dependent receptor/neurotransmitter systems will not be further described in the present review, it is important to emphasize that incoming stimuli, transduced through the glutamatergic system via NMDAR-regulated Ca^2+^ influx and its downstream effects, shape the entire synaptic framework, not just glutamate receptors. Stimulus-triggered Ca^2+^influx via NMDARs is therefore critical for the neural plasticity required throughout an organism’s life to adapt to environmental changes. While this NMDAR-mediated Ca^2+^signaling is best studied in the context of real-time CNS neural plasticity [94], it also contributes to the lifetime cellular epigenetic changes occurring in all cells of the organism, not just CNS cells. Consistent with a unifying theory, excessive activation of NMDARs has been involved in the pathogenesis of diseases in various peripheral organs [95].

## 5. Glutamate, NMDARs, Cognition and Mood Regulation

While ketamine and its S-enantiomer esketamine are effective in relieving depression, they cause dissociation in the majority of patients at the doses currently used for MDD [96]. We have reported that esmethadone, a low potency NMDAR uncompetitive antagonist, improves symptoms of depression, including subjective cognitive impairment, in patients with MDD without inducing euphoria, dissociation, or other perceived psychoactive effects [31,32]. Relief from depression without dissociative effects was demonstrated with other NMDAR uncompetitive antagonists, such as the dextromethorphan-bupropion combination [38].

Experimental evidence suggests that NMDAR uncompetitive antagonists may decrease depressive-like behavior by blocking the selectivity pore of NMDARs during resting membrane potential [23]. The improvement in the depressive phenotype by molecules that tonically block the NMDAR has led to the hypothesis that the depressive phenotype may be caused by NMDAR hyperactivity at resting membrane potential [28]. As previously discussed, the depressive phenotype, under certain circumstances (e.g., bereavement or chronic unpredictable stress), is “normal” and evolutionary favorable. This phenotype can also be voluntarily induced by intentionally focusing on a meaningful subject, e.g., during religious or political ceremonies, other solemn events, or even when focusing deeply on a matter of interest. This “normal” depressive phenotype, resembling “rumination”, a known component of pathological depression, may be driven by the same molecular mechanism: chronic NMDAR hyperactivity at resting membrane potential within relevant neural circuits. This hyperactivity leads to a tonic increase in Ca^2+^ influx in neurons within these circuits. The ability to maintain attention on a specific topic (e.g., during academic tasks) and the inability to shift focus (e.g., during pathological rumination) may share a similar molecular basis. The former is voluntary and physiological, while the latter is involuntary and pathological. The underlying mechanism in both cases may be the tonic hyperactivity of NMDARs in the “hot spot” of neurons within relevant neural circuits. In summary, both the physiological depressive-like phenotype and the MDD phenotype may be caused and maintained by hyperactivation, physiological or pathological, respectively, of NMDARs at resting membrane potential in relevant neural circuits.

Patients with MDD suffer not only from depressed mood but also from cognitive impairment, lack of motivation, and “rumination”. These symptoms may reflect an underlying impairment in neural plasticity. Specifically, the molecular mechanisms that enable circuity modifications necessary for learning new concepts and for overcoming fixed thoughts, are disrupted.

As previously noted, chronic excessive Ca^2+^ influx via hyperactive NMDARs at resting membrane potential inhibits the production, transportation, and assembly of synaptic proteins [23,28,39,40] (Figure 1). From a psychopharmacological perspective, NMDAR uncompetitive antagonists can downregulate excessive Ca^2+^ influx, thereby restoring homeostatic amounts of synaptic proteins. This restored homeostasis allows neural plasticity to resume efficiently in circuits relevant to MDD, reversing impairments in motivation and cognition and other symptoms characteristic of MDD, such as fixed thoughts (rumination) [28]. In animal models of depressive-like behavior, uncompetitive NMDAR antagonists have been shown not only to reverse depressive-like behavior but also to restore spinogenesis [11,13]. From an evolutionary standpoint, memories (neural circuits connected by synapses) may persist or dissipate based on their relevance to survival, in a balance between LTP and LTD. Synapses that encode experiences with a strong positive or negative impact on survival are preferentially maintained. In contrast, memories lacking significant survival relevance are formed with weaker synapses that more easily disappear [97]. The brain forms memories by decoding environmental stimuli that reach neurons via receptor cells. The essential function of all organs and tissues is to support and protect the brain. From the brain’s perspective, the body may be considered part of the external environment; however, stimuli originating from the body hold special importance because they provide information directly related to the survival of the brain. Therefore, the brain has a clear preference (selectivity) for memorizing experiences that originate from receptors collecting input from bodily organs and tissues. This preference for internal stimuli is hierarchically ranked among organs and tissues: for instance, chest pain, potentially originating from the heart, is likely more impactful on survival than muscle aches in the limbs, which are more likely to be interpreted as benign. Therefore, sudden, severe, and unexplained chest pain will integrate into existing circuits and trigger more structured memory compared to muscle aches after intense exercise. Selective sensory memories are not only useful for making predictions that enhance the probability of species survival but also direct and refine motor circuits that determine evolutionary behaviors and functions. Motor memories (remodeling of spines and development of new spines and connections in motor circuits) form when motor tasks are performed as predictions that may enhance species survival. Overall, memory formation (remodeling of synaptic framework and neural connections), like other characteristics of living organisms, has evolved to favor LTP of circuits with relevance for individual/species survival, rather than depleting resources by indiscriminately memorizing all incoming stimuli or memorizing indiscriminately all motor circuits [98].

Memory is formed through the creation and maintenance of synapses between neurons, establishing survival-relevant circuits based on stimuli reaching receptor cells. Glutamate is the primary neurotransmitter released when receptor cells are activated by external stimuli. Within presynaptic neurons, glutamate is stored in vesicles, and the size and number of these vehicles are determined by the type, intensity, and frequency of sensory stimuli reaching the sensory cells. When the density of glutamate-containing vesicles reaches a certain threshold, one or more vesicles fuse with the presynaptic membrane, even in the absence of a depolarizing stimulus. This fusion determines the release of glutamate into the synaptic cleft and subsequent activation of postsynaptic glutamate receptors. Once glutamate binds to its receptors, as previously described, Ca^2+^ influx via NMDARs modulates the synaptic framework, influencing the formation and maintenance of synaptic spines [14]. Pathological hyperactivation of NMDARs at resting membrane potential disrupts synaptic protein homeostasis and impairs neural plasticity. Uncompetitive NMDAR antagonists restore synaptic protein availability and thus neural plasticity by blocking the channel pore [11,13,23].

In a dynamic, real-life context, where multiple stimuli occur over time, each stimulus triggers pre-synaptic glutamate release followed by postsynaptic Ca^2+^ influx via NMDARs. This influx leads to downstream effects, including epigenetic changes such as modifications in protein subunit translation, production, transport, and assembly. Additionally, downstream effects include changes in receptor expression at synapses and changes in the production and release of neurotrophic factors.

A key concept is that NMDAR-mediated Ca^2+^ signaling modifies the synaptic framework, resulting in a different number of quanta of Ca^2+^ influx with each subsequent stimulus. Therefore, each new stimulus encounters a modified synaptic receptor framework, leading to a different number of quanta of Ca^2+^ entering the cell. This variation in Ca^2+^ quanta will determine a different pattern of downstream changes compared to the previous stimulus. Thus, the same stimulus can lead to different patterns of gene activation due to the difference in the number of quanta of Ca^2+^ entering the cell via an ever-changing framework of AMPARs and NMDARs at the synaptic “hot spot“. The coordinated opening and closing of NMDARs and AMPARs are pivotal for memory formation: while NMDARs serve as the actual memory decoders, AMPARs function as electrical switches for NMDARs, acting as dimmers/enhancers at graded resting membrane potential and as on/off switch during action potentials. At action potential, the AMPAR is “on”: all AMPARs bound by glutamate briefly (1 ms) switch to the open conformation, causing the membrane potential to shift below the −55 mV threshold and rapidly move toward positive values due to massive, “fast” Na^+^ influx via AMPARs. This shift in membrane potential leads to Mg^2+^ disengagement from NMDARs, making them permeable in a subtype-specific manner. When low nanomolar concentrations of glutamate (or certain pathological NMDAR agonists or PAMs) are present in the synaptic cleft, GluN2D subtypes have the highest probability of Mg2+ disengagement and the highest probability for transition to the open conformation. Quanta of Ca^2+^ flow into the cell, imparting epigenetic direction by activating specific enzymes. The amount of Ca^2+^ quanta entering the cell via NMDARs serves as the epigenetic code for neural plasticity, interacting with the intracellular machinery of NMDARs. Between action potentials, a small percentage of AMPARs are activated by glutamate released by the presynaptic neuron, contributing to the grading of resting membrane potential, which ranges from −85 to −55 mV. A more positive graded membrane potential increases the likelihood of Mg^2+^ disengagement from NMDARs. At resting membrane potential, only NMDARs subtypes with lower affinity for Mg^2+^, longer opening time, and greater affinity for glutamate, such as GluN2D subtypes, are more likely to be in the open conformation and free of Mg^2+^. This higher probability is specific to NMDAR subtypes and increases with increasing concentrations of ambient glutamate and or PAMs, whether endogenous (e.g., quinolinic acid) or exogenous (e.g., gentamicin) [1,28,43,51].

In summary, under certain pathological conditions (e.g., MDD), uncompetitive NMDAR antagonists can be characterized not only as “activity-dependent” [29] but as “hyperactivity-dependent” and therefore NMDAR subtypes most sensitive to pathological activation (i.e., GluN2D) are more likely to be blocked [43,51]. This block of hyperactive receptors may restore synaptic protein homeostasis [23], reverse depressive-like behavior, and restore neural plasticity [11,13,29]. The MOD for MDD may be tonic hyperactivity of GluN2D receptors in neurons part of circuits relevant for MDD and the MOA of uncompetitive NMDAR antagonists is a selective block of these hyperactive GluN2D receptors [28].

## 6. The Endorphin System, Mood Regulation and Neural Plasticity

In this section, we discuss the role of the endorphin system in conferring sentience and relevance to stimuli and we attempt to explain how opioid receptors structurally and functionally interact with NMDARs, facilitating the preferential integration of stimuli into functional neural circuits based on their sentience.

The centrality of the endorphin system in physiological mood regulation is well established [99]. Dysregulation of this system has been implicated in mood disorders, including MDD [100]. From an evolutionary and survival perspective, the endorphin system plays a crucial role in evaluating the species-preserving impact and relevance of incoming stimuli by conferring positive (via MORs) or negative (via KORs) sentience to experiences. This system is essential for the ongoing formation of functional neural circuits (neural plasticity) by facilitating the preferential integration of circuits selected by experiences that promote species-preserving prediction-based activities. Additionally, the endorphin system, via both MOR and KOR pathways, provides well-known analgesic effects.

Evolutionarily favorable stimuli, such as those related to food, sex, and physical activity, stimulate neurons in the arcuate nucleus of the hypothalamus to release beta-endorphin. The released beta-endorphin binds to MORs, including those in the ventral tegmental area (VTA), thereby activating the reward system. This activation enhances mood, motivation, and cognitive processing, reduces stress, increases the pain threshold, and reinforces the overall experience [101]. By activating MORs, beta-endorphins enhance neural plasticity in circuits related to positive experiences crucial for species preservation, thereby reinforcing the repetitive pursuit of stimuli associated with food, sex, and physical activity.

The selective integration of neural circuits driven by beta-endorphin binding to MORs is believed to be mediated by the structural association of MORs with NMDARs. When beta-endorphins bind to MORs, they induce positive allosteric effects on structurally associated NMDARs [102,103]. This same molecular mechanism underlies addiction to exogenous opioids, such as morphine, which similarly exerts positive allosteric effects on NMDARs associated with MORs [104]. Based on this premise, the physiological, evolutionary favorable reward mechanisms and the pathological mechanisms of addiction share the same molecular foundations. These “selected” memories can be structurally and functionally explained by the positive allosteric modulation of NMDARs associated with MORs by opioids [103,105].

Aside from playing a role in selecting LTP for species-preserving evolutionary favorable behaviors, the endorphin system also plays a role in learning and memory of avoidant behaviors, which are equally important for preferential integration of experiences conducive to species-preserving prediction-based activities [106].

Evolutionarily unfavorable stimuli, such as those associated with actual or potential tissue injury, stimulate neurons in the arcuate nucleus of the hypothalamus to release dynorphins. These dynorphins bind to kappa opioid receptors (KORs), including those in the lateral habenula (LHb). This binding increases the pain threshold, reduces stress, and activates cognitive processing [107], similar to the effects of endorphin binding to MORs. However, in contrast to the euphoria produced by beta-endorphin binding to MORs, dynorphin binding to KORs induces dysphoria, leading to avoidant behaviors. While these behaviors are evolutionarily favorable for avoiding harmful stimuli, they can become pathological under certain circumstances, notably in the context of the depressive phenotype [100].

Dynorphins, by activating KORs that are structurally associated with NMDARs, enhance neural plasticity (LTP) in circuits memorizing negative experiences. This selective integration of negative experiences is evolutionarily advantageous, as it reinforces the avoidance of species-prejudicial stimuli, such as those associated with tissue damage. The integration of these experiences is crucial for wiring afferent circuits to efferent circuits that are involved in species-preserving, prediction-based avoidant activities.

Physiological and pathological avoidance behaviors share the same molecular mechanisms and represent forms of memory that can be structurally and functionally explained by PAM effects of endogenous (dynorphins) and exogenous (KOR agonists, e.g., levorphanol) opioids, with physiologically or pathologically enhanced Ca^2+^ influx via NMDARs, respectively [108].

The significance of the dynorphin system in mood regulation is further supported by the recent development of KOR antagonists as antidepressants. These novel antidepressant candidates antagonize the binding of dynorphins, thereby reducing dysphoria induced by KOR activation [109].

A recent study confirms that ketamine blocks hyperactive LHb NMDARs in depressive-like animals, underscoring the importance of the LHb as a hub for circuits relevant to mood regulation. This study highlights the brain region- and depression state-specific M OA of activity-dependent uncompetitive NMDAR antagonists. It suggests that lower doses of these agents may selectively target pathologically hyperactive NMDARs within circuits relevant to disease states, including but likely not limited to MDD [29].

The ability to form relevant structural and functional memory—integrating neural circuits to recall “negative” and “positive” experiences—enhances species-preserving behaviors. Endorphin-selected neural plasticity increases the likelihood of avoiding negative experiences and repeating positive ones by enabling the wiring of afferent and efferent circuits involved in species-preserving, prediction-based activities. The well-known Pavlovian experiment in dogs first demonstrated the conditioned wiring of sensory input to efferent circuits [68]; in non-experimental, physiological settings selective wiring is driven by experiences selected by sentience conferred by the endorphin system.

In summary, the endorphin-regulated preferential integration of positive and negative experiences into relevant neural circuits wires afferent and efferent circuits deputed to species-preserving prediction-based activities.

Methadone, a synthetic opioid, also acts as an uncompetitive NMDAR antagonist. This NMDAR antagonism likely contributes to its overall pharmacological effects [110,111,112,113]. Specifically, methadone’s NMDAR antagonistic activity may counteract the PAM effects of opioid receptor binding on NMDARs [104], which could explain its reduced addiction potential compared to other MOR agonists, evidenced by its use as a treatment for opioid use disorder spanning over many decades and across many countries.

Endogenous opioids, beta-endorphin, and dynorphins, similar to the exogenous opioids, morphine, and levorphanol, act as PAMs at NMDARs structurally associated with MORs and KORs, respectively. When endogenous endorphins are released by hypothalamic neurons or when exogenous opioids are administered, they increase the open probability of the NMDAR channels that are structurally associated with opioid receptors. This increase in open probability facilitates a greater Ca^2+^ influx when Mg^2+^ is disengaged from NMDARs. The endorphin-induced opening of NMDARs structurally associated with opioid receptors may be the common molecular basis for several phenomena, including the following: (1) selective memorization of evolutionary favorable incoming stimuli; (2) pharmacological tolerance to opioids, a form of learning involving changes in the receptor framework; and (3) addiction caused to exogenous opioids, due to NMDAR activation and formation of reward circuits [102,103,104,114].

In summary, learning, including the selective memorization of impactful stimuli and events, is modulated by endorphins through the structural and functional association between opioid receptors and NMDARs. This modulation is an evolutionary advantage, allowing organisms to selectively memorize positive and negative experiences that are crucial for making predictions and decisions favorable to species survival. The selective memory of pleasant and unpleasant events, facilitated by the binding of beta-endorphin or dynorphin to MORs and KORs, respectively, provides a molecular basis for “emotional intelligence”. This concept refers to the use of emotional cues in daily decision making. Emotional cues are structurally represented as neural circuits that integrate incoming stimuli with hierarchical species-preserving positive or negative relevance. By selecting which positive and negative experiences to integrate into the existing neural circuitry, the endorphin system influences risk assessment, social interactions, and moral and ethical judgments. The endorphin-selected neural circuitry underpins a molecular basis for sentience as a driver of neural plasticity.

## 7. Targeting NMDARs for the Treatment of Major Depression

Uncompetitive NMDAR antagonists are activity-dependent molecules because they can only block NMDAR channels when they are in the open conformation and free of Mg^2+^. As the proportion of NMDAR channels in the open conformation and free of Mg^2^ increases (e.g., when the membrane potential shifts from −85 mV towards a more positive value), the activity of uncompetitive NMDAR antagonists also increases. Ketamine, a relatively high-potency uncompetitive NMDAR antagonist, blocks a relatively higher percentage of NMDARs compared to less potent NMDAR blockers such as dextromethorphan and esmethadone [43]. When administered at doses used for MDD (0.5 mg/Kg), ketamine is a rapid-acting antidepressant, with effects persisting well beyond detectable serum levels. At higher doses, ketamine induces anesthesia. Lower potency uncompetitive NMDAR antagonists, such as dextromethorphan and esmethadone, do not cause dissociative effects at antidepressant doses. This is because they block a lower percentage of NMDAR channels in the open conformation and are free of Mg^2^, reducing the likelihood of interference with NMDARs during physiological action potential. The proportion of NMDAR channels in the open conformation and free of Mg^2+^ is increased in pathological states involving chronic excitotoxicity [54]. Therefore, low potency uncompetitive NMDAR antagonists may exert selective activity during pathological conditions, blocking NMDAR channel pores only when necessary.

The complexity of NMDARs apparently provides the opportunity for different potential drug targets, with both therapeutic and pathological implications, including competitive agonists or antagonists at glutamate or glycine binding sites, non-competitive antagonists acting in the proximity of the glutamate and glycine sites, or PAMs or NAMs. However, the critical role of NMDARs in physiological neural plasticity may de facto limit the therapeutic MOA of pharmaceutical candidates to low potency uncompetitive antagonists, which exhibit only hyperactivity-dependent effects and no effects on the physiological activity of NMDARs. At therapeutic doses for MDD, these low-potency uncompetitive antagonists are active primarily only during pathological states when an abnormally high proportion of NMDAR channels is in the open conformation and free of Mg^2+^ (Figure 1). In summary, the activity-dependent effects of low-potency uncompetitive NMDAR antagonists allow for selective targeting of pathologically hyperactive NMDAR channels without meaningfully affecting physiological glutamatergic activity. Additionally, different NMDAR subtypes vary in their affinity for Mg^2+^, glutamate, and competitive agonists or PAMs (e.g., quinolinic acid) [1,43,51]. Chronic excitotoxicity, driven by low levels of glutamate or other agonists or PAMs synthesized in pathological conditions, may preferentially hyperactivate GluN2D-containing NMDAR subtypes, making these subtypes the preferential target of low-potency uncompetitive antagonists [28].

The reversal of depressive-like behavior by uncompetitive NMDAR antagonists in preclinical models appears to involve synaptic protein restoration through BDNF-dependent mechanisms [11,13,23]. Ketamine, a dissociative anesthetic, has been the prototype of this novel class of uncompetitive NMDAR antagonist antidepressants since its initial demonstration of antidepressant efficacy [19,33]. Esketamine, the S-enantiomer of ketamine, has replicated the effects of the racemic molecule and was approved by the FDA for TRD in 2019 [115]. Uncompetitive NMDAR antagonists with antidepressant efficacy are a relatively recent class of molecules. The dextromethorphan-bupropion combination has also shown efficacy for MDD in Phase 2 and Phase 3 clinical trials [37,38] and was FDA-approved for the treatment of MDD in 2022. Esmethadone (REL-1017), a novel low-potency uncompetitive NMDAR antagonist, demonstrated antidepressant effects at both tested doses (25 and 50 mg daily) in a Phase 2 trial in patients with inadequate response to standard antidepressants [31], without producing the euphoric opioid-like or dissociative ketamine-like effects. While esmethadone did not meet its primary endpoint in a Phase 3 trial, it showed efficacy in the subgroup of patients with severe depression [32]. The selectivity of esmethadone for severely depressed patients may be due to its activity-dependent mechanisms: only patients with pathologically hyperactive NMDARs in neurons part of circuits relevant to MDD may be susceptible to its effects.

Results from studies of uncompetitive NMDAR antagonists conducted in silico [43], in vitro [43,51], in vivo [11,12,13], and in humans [31,38,115] support the emergence of a new class of antidepressants with a common hypothesized antidepressant MOA [28]. Recent experimental results showed that ketamine specifically blocks NMDAR currents in lateral habenula LHb neurons in depressive-like mice but not naïve mice, suggesting that its action occurs only within pathological circuitry relevant to the depressive phenotype [29].

The “homeostatic hypothesis”, which posits that blocking NMDARs at the resting membrane potential restores physiological neural plasticity via BDNF-dependent mechanisms, is well supported [11,23,28] and is gaining wider acceptance [41,42]. As new experimental data are gathered [29], this hypothesis gains plausibility. The full hypothesis suggests that the restoration of neural plasticity in both preclinical models and patients may result from uncompetitive NMDAR antagonism, with a preference for targeting tonically hyperactive GluN2D subtypes [28].

The proposed MOA of uncompetitive NMDAR antagonists is further supported by the clinical phenotype of patients with MDD. These patients experience not only depressed moods but also a lack of motivation and cognitive deficits, which are believed to stem from impaired neural plasticity in specific brain areas and circuits, such as the medial prefrontal cortex (mPFC) and its associated networks. These cognitive symptoms are especially evident in patients with severe MDD.

In preclinical models, uncompetitive NMDAR antagonists have been shown to activate the mPFC [11,13,23]. The ability of uncompetitive NMDAR antagonists to revere depressive-like behavior and restore neural plasticity in animal models was first demonstrated with dizocilpine (MK-801) [116]. However, the uncompetitive NMDAR block exerted by MK-801 resulted in a profound alteration of physiological NMDAR activity, likely due to the high potency of this molecule, resulting in severe adverse effects that have precluded its clinical application. The antidepressant efficacy of ketamine in humans was first observed over 20 years ago [19]. Subsequently, in murine models, ketamine was found to reverse depressive-like behavior, restore synaptic proteins, and enhance synaptic spines [13,23]. These findings observed with ketamine have been replicated with esmethadone [11]. Since neural plasticity is known to be driven by NMDAR activation [1,14], the restoration of neural plasticity by an NMDAR blocker with dissociative effects, such as ketamine, at first was viewed as counterintuitive. To resolve this apparent paradox, the “disinhibition hypothesis” was proposed. According to this hypothesis, ketamine preferentially blocks NMDARs on GABAergic inhibitory interneurons, thereby reducing overall inhibition, which in turn disinhibits excitatory neurons and enhances excitatory synaptic transmission in the mPFC [35]. This hypothesis assumed that the psychotomimetic effects of ketamine were necessary for the restoration of neural plasticity observed in animal models of depressive-like behavior and for its antidepressant effects in humans. Thus, the disinhibition hypothesis served as an early attempt to reconcile the dissociative effects of ketamine with its antidepressant activity. However, it is now believed that these two effects (dissociative and antidepressant) can be experimentally and clinically separated [36]. Furthermore, the efficacy of uncompetitive NMDAR antagonists for MDD without psychotomimetic effects [31,37] further weakened the foundation of the disinhibition hypothesis, which lacked strong experimental support.

A more recent hypothesis explaining the effectiveness of NMDAR antagonists in MDD has emerged from decades of experimental work. Under normal physiological conditions, tonic Ca^2+^ influx through NMDARs regulates synaptic protein synthesis by activating eukaryotic elongation factor-2 kinase (eEF2K, also known as CaMKIII). This kinase phosphorylates eEF2, halting synaptic protein translation [39,40]. In preclinical models of depressive-like behavior, uncompetitive NMDAR antagonists have been shown to restore synaptic protein synthesis by downregulating excessive postsynaptic Ca^2+^ influx via NMDARs during resting membrane potential [23,27,28] (Figure 1). Excessive Ca^2+^ influx in the postsynaptic neuron during the resting membrane potential interferes with the local availability of synaptic proteins required for effective neural plasticity via hyperactivation of CaMKIII [23,24,25,26,27,28,39,40,41,42]. Local availability of synaptic proteins at synaptic spines is necessary for efficient physiological neural plasticity. When synaptic proteins are unavailable, due to NMDARs hyperactivity and increased Ca^2+^ influx with hyperactivation of CaMKIII, neural plasticity is impaired. As already mentioned, this deficiency can result in physiological depressive behavior, such as during bereavement, which is considered an “appropriate” response. However, prolonged unavailability of synaptic proteins in key neural circuits, such as the mPFC, leads to hypoactivity in these regions, contributing to the MDD phenotype, which is characterized by not only depressed mood but also impaired cognition and motivation. Uncompetitive NMDAR antagonists exert an activity-dependent block at the NMDAR channel pore (Figure 1) and reduce excessive NMDAR-mediated Ca^2+^ influx [43]. This downregulation of tonic NMDAR activity restores physiological neural plasticity (Figure 1) [28], ultimately resolving the depressive phenotype [31,38,115].

Furthermore, low potency uncompetitive NMDAR antagonists, such as esmethadone and dextromethorphan, administered at therapeutic doses for MDD selectively block NMDAR subtypes that are in the open conformation and free of Mg^2+^ during resting membrane potential (Figure 1). In contrast, higher potency uncompetitive NMDAR antagonists, like ketamine, at antidepressant doses currently in use (0.5 mg/kg), also block NMDAR channels in the open conformation and free of Mg^2+^ during action membrane potential. This broader blocking action can result in a dissociation between incoming environmental stimuli and neuronal responses, manifested clinically as dissociative and psychotomimetic effects, commonly seen after ketamine and esketamine administered at antidepressant doses in current use.

When glutamate concentration in the synaptic cleft is in the low nM range, such as around 40 nM, NMDAR subtypes with higher sensitivity to glutamate and lower affinity for Mg^2+^ are preferentially activated. These subtypes have a higher probability of remaining open and free of Mg^2+^. Notably, GluN2D subtypes have the highest affinity for glutamate and low affinity for Mg^2+^ [1,43,80,117]. In the presence of esmethadone, these hyperactive, conformationally open GluN2D subtypes are preferentially blocked [43]. This preference for GluN2D subtypes is shared by other NMDAR antagonists [118]. GluN2D subtypes are also particularly sensitive to endogenous PAMs, such as the inflammatory intermediate s quinolinic acid, exogenous PAMs (e.g., gentamicin, NMDA), and endogenous or exogenous competitive agonists [51]. Multiple NMDAR PAMs or NAMs may increase or decrease, respectively, the probability of Ca^2+^ influx via NMDARs [1,43,51]. Quinolinic acid, a kynurenine downstream metabolite, preferentially increases the open probability for GluN2D subtypes, whereas GluN2C subtypes are relatively insensitive to its effects [43,51,119].

In summary, excessive ambient glutamate, as well as endogenous or exogenous agonists and PAMs, can pathologically increase Ca^2+^ influx via NMDARs, leading to chronic excitotoxicity, which contributes to the MDD phenotype and potentially other neuropsychiatric and neurodegenerative conditions, depending on the affected neural circuitry [54].

Esmethadone has approximately 10-fold less affinity for NMDARs compared to ketamine [43]. The potency and effects of uncompetitive NMDAR antagonists are determined by both their affinity for the binding site within the channel pore and their degree of trapping [120]. Mg^2+^, the physiological blocker of NMDARs, has a subtype-specific affinity and low trapping, as its engagement within the receptor pore is primarily dependent on membrane potential and Mg^2+^ concentration. Compared to MK-801, memantine has moderate affinity for the PCP site within the NMDAR channel pore, similar to ketamine. However, memantine exhibits lower trapping compared to ketamine and esmethadone. Memantine lacks lack efficacy for MDD but is approved for Alzheimer’s disease. Ketamine, on the other hand, has moderate affinity compared to MK-801, similar to memantine, but in contrast with memantine, it has higher trapping, which may contribute to its efficacy for MDD and may contribute to its dissociative side effects. Esmethadone, with a lower affinity for NMDARs compared to ketamine and memantine, but with ketamine-like trapping (which is higher relative to memantine), may be effective for severe MDD, without inducing dissociative side effects [31,32,51].

High trapping may be crucial for therapeutic efficacy in MDD, while low affinity prevents dissociative side effects. In contrast, uncompetitive NMDAR antagonists with low trapping, such as memantine, do not cause dissociation despite moderate receptor affinity, similar to ketamine.

Thus, low affinity coupled with high trapping may be an ideal characteristic for uncompetitive NMDAR antagonists that are well-tolerated and effective as antidepressants without dissociative side effects [28,43]. Esmethadone and dextromethorphan may have optimal affinity and trapping properties, making them potentially effective antidepressants without dissociative effects.

In summary, neural plasticity is regulated by glutamatergic signaling via NMDARs [1,14]. While both phasic and tonic receptor activities can be blocked by uncompetitive NMDAR antagonists, low potency NMDAR antagonists, at concentrations therapeutic for MDD, preferentially block NMDAR during tonic activity, at resting membrane potential. A higher potency block is required to block NMDARs during phasic activation, achieved by higher affinity and higher trapping NMDAR antagonists like ketamine, or achieved at very high concentrations of low-potency (low affinity, low trapping) NMDAR antagonists such as dextromethorphan. However, with low-affinity uncompetitive antagonists, such as esmethadone and dextromethorphan, and with low trapping drugs, such as memantine, the high concentrations required for NMDAR block during action potential may not be achieved in humans, due to other dose-limiting side effects, e.g., nausea and vomiting for esmethadone [121,122,123].

Given the complex relationship previously described between glutamate, NMDARs, and the endogenous opioid system, a third hypothesis, the “opioid agonist hypothesis” has been proposed to explain the antidepressant effects of ketamine, dextromethorphan, and esmethadone [45,46]. This hypothesis is based on the observation that opioid receptor blockade with antagonists attenuates the antidepressant effects of ketamine in humans and in animal models of depression [124,125]. However, while a functioning opioid system is required for the antidepressant effects of ketamine, these effects are not due to direct mu opioid agonism [124]. In vitro and in vivo evidence indicates that even opioid-derived uncompetitive NMDAR antagonists with opioid affinity lack meaningful opioid-like agonist effects in vivo and in humans [31,46,122,123,126,127]. Thus, while direct opioid agonist effects do not contribute to the antidepressant action of NMDAR antagonists, the interplay between the NMDARs and the endorphin system helps explain why a functioning endorphin system is necessary for their antidepressant effects, as described by Klein and colleagues [124].

## 8. Consciousness of Self and Individuality

Consciousness of self, at any given moment, results from the real-time integrated processing of environmental and bodily stimuli within and into the individual’s unique neural circuitry. As discussed in prior sections, the integration of stimuli is regulated by ionic, electrochemical, and molecular activity at NMDARs.

The rubber hand illusion (RHI) is an example of stimulus processing and integration in the individual’s neural network. The RHI is an experiment in which tactile stimuli applied to a person’s real hand (hidden from view) are synchronized with corresponding visual stimuli observed on a rubber hand placed where the real hand would normally be. This creates the illusion that the rubber hand is part of the individual’s body (self). Interestingly, another study demonstrated that subjects can experience parts of the environment, such as the rubber hand, as part of their own body, not only through synchronous visual and tactile stimuli (RHI) but also through visual stimuli alone. In this case, the illusion was produced by visual stimuli coupled with predictions of tactile sensations, even in the absence of actual tactile input [128]. Virtual reality has also been used to further understand of self-consciousness [129].

Stimuli, such as the tactile and visual stimuli in the RHI, lead to receptor cell shifts in membrane potential and presynaptic glutamate release, activating ionotropic receptors. The NMDAR-mediated influx of Ca^2+^ quanta regulates the synaptic framework and integrates incoming stimuli into neural circuits. At any given moment, each individual’s neural circuitry generates a real-time perception of self. NMDAR-mediate neural plasticity is the mechanism for perceiving the environment and for making predictions, including the perception and prediction of “self”.

Individuality is structurally and functionally represented by the unique neural circuitry of each individual, which is already distinct at birth, as signaled by known phenotypic differences between genetically identical twins. Neural plasticity, the brain’s ability to constantly adapt and reorganize, is shaped from conception onward by the individual’s exposure to environmental stimuli. This exposure leads to Ca^2+^ quanta influx via NMDARs, regulating the brain’s wiring. In humans and other animals, the endorphin system exerts PAM effects on NMDARs, adding sentience and evolutionary hierarchy to neural circuitry. Neural plasticity is a continuous process that never stops during an individual’s lifetime. Therefore, individuality is not a static trait. Individuality evolves continuously, as new stimuli are integrated into the individual’s circuitry. Each individual’s neural circuitry is not only distinct from others’, including an identical twin, but it is also different from its previous state, just moments ago. For instance, in the absence of significant events, these circuitry changes may be slight and non-meaningful. However, if a dramatic life-threatening event should occur, e.g., a myocardial infarction, many synapses of the individual’s neural circuitry will change. Within seconds, these synaptic changes will have changed the individual’s emotions, behaviors, beliefs, and predictions in a very meaningful way.

At any given moment, consciousness of self consists of the integration of incoming stimuli within and into the individual’s circuitry. Simple and complex sensory experiences, as well as motor activities, can ultimately be traced back to NMDAR-mediated Ca^2+^ quanta influx and its downstream effects on synapses and neural circuits.

## 9. Summary and Conclusions

The studies we presented support the primary role of Ca^2+^ influx via NMDARs in the pathophysiology of mood disorders. Importantly, they allow us to propose the hypothesis that Ca^2+^ quanta via NMDARs serve as the epigenetic code for neural plasticity. These Ca^2+^ quanta regulate the membrane expression and functionality of neurotransmitter receptors, including NMDARs and AMPARs, in particular those localized at the synaptic “hot spot” in postsynaptic neurons.

By activating specific downstream enzymatic pathways, Ca^2+^ quanta trigger epigenetic mechanisms that control the homeostasis of synaptic proteins. Balanced availability of synaptic proteins is necessary for stimulus-determined processes such as transcription, receptor trafficking, synaptic spine plasticity, and ultimately neural plasticity.

The plasticity of neural circuits is driven by the integration of incoming stimuli, and NMDAR-mediated Ca^2+^ influx dictates the equilibrium between LTP and LTD. Neural plasticity maintains an adaptive state for neural circuits to incoming stimuli and is crucial for synaptic scaling, a process largely mediated by AMPARs. Importantly, disruptions in this mechanism, as observed experimentally in the chronic-stress-induced depressive phenotype, highlight the potential pathological effects of tonically hyperactive NMDARs, in particular GluN2D subtypes, at resting potential. This excessive NMDAR-mediated Ca^2+^ influx results in a reduction in synaptic protein synthesis, impairing neural plasticity and causing cognitive and motivational deficits, in addition to the emotional and behavioral changes associated with the MDD phenotype.

Psychopharmacological advances are shedding light on the interplay between the glutamatergic and endorphin systems in the modulation of mood and behavior. Endorphins, such as beta-endorphins and dynorphins, act as PAMs of NMDARs and play a pivotal role in strengthening emotionally charged neural circuits through LTP. The endorphin-NMDAR interaction underlies the molecular basis of LTP in circuits related to reward and evolutionary behaviors, providing a molecular mechanism for explaining addiction.

Furthermore, this unifying hypothesis links foundational theories from evolutionary biology, such as Darwin’s work on species preservation, to modern understandings of NMDAR function and neural plasticity. Concepts, such as the “Proustian moment”, where specific sensory stimuli evoke vivid emotional memories, can be explained through stimulus-triggered, NMDAR-mediated synaptic strengthening of the individual’s neural network shaped by lifetime experiences. The theory suggests that this process not only governs basic survival mechanisms but also shapes complex behaviors and emotions, bridging evolutionary perspectives with modern psychopharmacology.

In summary, this review presents a unifying theory that emphasizes the pivotal role of NMDAR-mediated quantal Ca^2+^ influx in neural plasticity and consequently in determining moods, thoughts, behaviors, and consciousness of self. By highlighting the interplay between the ionotropic glutamatergic systems, synaptic proteins, and the endorphin system, this hypothesis provides an ionic, electrochemical, and molecular framework for understanding human emotions, behaviors, and individuality. It also bridges evolutionary biology and neuroscience, offering new insights into the pathophysiology and therapy of neuropsychiatric disorders.
